# Book Reviews

**Published:** 1815-12

**Authors:** 


### CRITICAL ANALYSIS

### OF RECENT PUBLICATIONS

IN THE

### DIFFERENT BRANCHES OF PHYSIC, SURGERY, AND MEDICAL PHILOSOPHY.

---

*Pharmacopœia Collegii Regalis Medicorum Londinensis*, M.DCCC.IX. Editio altera.

*The Pharmacopœia of the Royal College of Physicians of London*, M.DCCC.IX. Translated into English, with Notes, &c. by RICHARD POWELL, M.D. The Third Edition.

WE have put these two articles together, because we neither presume to question the latinity of that learned body, nor the faithfulness of Dr. Powell's translation. We may, however, be allowed to ask of some of our learned readers, how long it has been customary to put the sign of the ablative (^) over those terminations in *a* of the first declension which have a preposition prefixed, as *in Angliâ.* We admit the custom may be traced back as far as the edition of 1746; we believe, however, it was omitted in 1787, and can see no necessity to mark a *case* which has its preposition before it.

By Dr. Powell's preface to the *third* edition of his translation of the London Pharmacopœia, the intention of the College seems to have been rather to correct the former edition, than to compile a *new* dispensatory. We are told "that the alterations adopted refer," first to some important processes to which reasonable objections have certainly been urged," among which the *antimonium tartarisatum* is given as an example—that the introduction of new articles has been "sparingly adopted"—that some things are restored which had stood in the Pharmacopœia of 1787—and that "a very few omissions from the last edition" have been made.

We have likewise been informed that the plan first proposed was nothing less than to establish, through the sanction of Parliament, a *national* Pharmacopœia for the whole of the British dominions, in which there should be at least an uniformity in prescriptions and arrangement, and that the Colleges of Edinburgh and Dublin should be invited to take their share in forwarding this most laudable and necessary design.

The great discordance between the three British dispensatories has long been a subject of complaint; and, although often noticed, there yet seems no serious disposition in either of the Colleges to associate with the other, in order to accomplish complish a reformation so important to the profession as well as to society at large. The examples of trifling disparity are too numerous to be noticed: it will be sufficient to mention only two—the *Empl. Lyttæ* and the *Confectio Aromatica*. According to the London College, the proportion of cantharides in the whole composition is equal to $\frac{2}{7}$; that of the Edinburgh is $\frac{1}{4}$; and the Dublin College prescribe $\frac{3}{10}$. These plasters are at equal variance in their consistence, price, and in the number and proportions of their ingredients. If lard be requisite in one prescription, why should it be omitted in the others? and why should the proportions of suet, wax, and resin, be subject to such capricious variation? As to the Confectio Aromatica, or, which is meant to be the same, the Electuarium Aromaticum, nothing can be more dissonant than the formulæ adopted by the Colleges of London, Edinburgh, and Dublin, particularly the two former. How embarrassing it must be for the patient and apothecary to find a difference in the smell, colour, taste, and other sensible qualities, of any article which may be prescribed in one part of the country and made up in another.

By the translator's preface we are further informed, that a Pharmacopoeia being of an ephemeral nature, requiring certain changes after no very long duration, a revision of the present will be demanded at no distant date; that Parliament are now employed in fixing a national and uniform standard for weights and measures; and that certain alterations in these are expected to take place which it will be for the College to examine, and determine whether to claim a peculiar exemption for the compounder of medicines, or agree to a general adoption of one standard for all purposes. These and other matters detailed in Dr. Powell's preface, incline us to regard the present edition of the London Pharmacopœia as a temporary performance, to correct the more palpable omissions and mistakes. We consider, therefore, that the original plans of the Committee, and, consequently, of the College, have been unexpectedly postponed, but not abandoned; and that the present correction will soon be followed by a more acceptable and finished volume of medical prescriptions.

On the subjects of weights and measures, we cannot, however, help offering a-fresh the hint contained in the History of *Pharmacopœias*, which will be found in our Journal soon after the appearance of the first edition of the works before us.* To save our readers the trouble of turning to it, we shall transcribe a short paragraph.

* See London Medical and Physical Journal, vol. xxii. p. 265.

"Respecting weights, I can see no reason why we should not confine ourselves, like most experimental philosophers, to grains altogether, or at least omit all the intermediate divisions from them to ounces or even pounds. By these means we might avoid all the Roman names, as well as technical characters. In the measures the word *congius* is objectionable, as marking a Roman measure different from our gallon. I can see no objection to gallo or gallonium, as the Pharmacopœia of 1745 had pinta for our pint."

After these few remarks, we shall proceed to a cursory examination of the principal changes that have been adopted, and occasionally turn to such other parts as deserve our notice, and may afford information to our readers.

*Acidum Aceticum.*—The title here is very objectionable, since there is no guide to prevent this preparation from being confounded with the *concentrated* acid, the radical vinegar, the acidum aceticum Ph. Dub., and the acidum acetosum forte of the Edinburgh College, all of which are synonymous. The Committee were long ago advised to add the word *mitius* or *dilutum*, or to say at once, Acetum purificatum. As the concentrated acid had been received in the Pharm. L. of 1787, we cannot reconcile ourselves to placing acetum distillatum as a synonyme with the title employed by a sister college for an acid of such superior power.

*Acidum Muriaticum.*—Here, and in many other of the formulæ, there is an approximation towards perfection in ascertaining the necessary proportions of the ingredients; there is a trifling change in the quantity of sulphuric acid, and we are told by Dr. Powel, that this change is founded on Vauquelin's experiments. Certainly there must be some allowance made for the state of dryness to which the muriate is reduced, for it is not ordered, as by the Edinburgh College, to be first made red-hot.

*Acidum Nitricum.*—The principal reason assigned for the great excess of the sulphuric acid for this process is, that the acidulous sulphate being more soluble than the neutral sulphate, it may be removed by warm water, and the glass retort thereby saved. The manufacturers who prepare this acid in such large quantities, use iron pots and no excess of acid. Other advantages are supposed to result when the sulphuric acid predominates and glass vessels are employed; the muriatic acid is readily separated in the beginning of the process, and the sulphuric acid rises only by mismanagement of the fire towards the end of the operation, otherwise the nitric acid, after the first running, is very pure. If this statement be correct, we should prefer the changing of the receiver during the process, to a re-distillation of the acid from nitrate of potass.

Objections might be made to other acids, but we shall now offer a few remarks on the alkalies.

*Ammoniæ subcarbonas.*—In the last edition, the quantity of chalk for this preparation was beyond even what was ordered in the Pharmacopœia of 1787, being the same as that of 1745. A trifling excess of the carbonate of lime is, however, of very little consequence; but there must be some difference between creta and creta *exsiccata*. This being the case, the proportion now prescribed is not so much diminished as it appears to be, and it will still bear a further reduction. The prefix *sub* is certainly very proper, but we are no advocates for such trifling alterations, and are doubtful whether such a very perspicuous nomenclature and faultless arrangement for any work of this nature can ever be acquired as will be generally acceptable.

*Liquor Ammoniæ.*—Lime is a substance of no great value, and a slight excess of it in this and some other processes can be of no moment. In almost every case, it is advisable to use rather more than what is sufficient for the purpose of decomposition or combination, especially as lime is not uniformly pure.

*Liquor Ammoniæ Carbonatis.*—Nothing appears more simple than this formula, just to mix one thing with another; but, as the carbonate of ammonia involves a previous operation, it will probably be in the end more economical to detach it at once from the muriate in the old way, by impure carbonate of potash and water. A slight degree of heat, perhaps about 90°, and agitation, will be requisite to perfect the solution.

*Potassæ Subcarbonas.*—We have here a choice of two ways of procuring the same article, although not in an equal state of perfection, nor with the same economy. We admit, with the College, "purior præparari potest ex tartaro," but there are some other instances where an alternative might also have been given with more propriety, and the two following processes will serve as examples.

*Potassæ Carbonas* and *Sodæ Carbonas.*—After instructing the operator how to accomplish the object by means of the subcarbonate of ammonia, as described in the text, the usual well-known and more economical method of applying carbonic acid to the solution of the alkaline subcarbonates should have been added. There is a mistake in Dr. Powell's note on sodæ carbonas. At the end, he should have said the crystals will still render vegetable blues *green;*—"will still *redden* vegetable blues" is, probably, an oversight.

*Potassæ Tartras.*—This, and many other neutral salts, seem to crystallize more perfectly when the alkali or base rather

 exceeds exceeds saturation. The Committee for the Pharmacopœia of 1809 were then recommended to add more of the sub-carbonate of potass to this very formula, as well as to the following, viz.

*Soda Tartarizata.*—Some objections are urged against this name, but they appear unfounded. The compound is formed of a substance called tartar with another called soda, and the proportions are definite. Any other title could not convey a more correct idea of the real nature of this salt.

*Calcis Murias.*—This salt and its solution are now, for the first time, admitted into the London list of remedies. Besides the form here inserted, of preparing it from the residuum of subcarbonate of ammonia, an article which, probably, no apothecary ever makes, a saturated solution of chalk or common oyster-shell in muriatic acid, and evaporated to perfect dryness, might have been named as a substitute, especially as such a process requires no great share of practical knowledge in the operator.

*Antimonii Oxydum.*—The synonyma enumerated by Dr. Powell are certainly at great variance with each other. Thus the antimonium calcinatum of P. L. 1787, and the calx antimonii of P. L. 1745, are perfectly inert; while the present oxydum, the crocus, and the antimonium vitrifactum, are intensely potent, although not altogether the same in their constituent principles. We do not conceive the necessity of including this preparation in any dispensatory, unless the intention be, by mixing it with phosphate of lime, to form a kind of James's Powder. Our experiments are not sufficiently matured to enable us to decide, whether this oxide is a subcarbonate of the metal, and whether an additional dose of carbonic acid to the solution of the alkaline subcarbonate would improve this preparation. If a pure oxide or protoxide be required, we presume that one more reasonable in respect to time and expense might have been devised; for, as we have already noticed, a prescription may possess the appearance of simplicity, and even elegance, when other processes ought to be pre-supposed as requisite, although not detailed in the prescription. Here the antimonium tartarisatum must, in the first place, be procured: if, therefore, the submuriate or pulvis algarothi of the Dublin College be more economical, we should certainly give it the preference, and substitute it for this formula.

*Antimonium Tartarisatum.*—This is universally acknowledged to be the most valuable improvement in the present edition of the London Pharmacopœia; and in whatever light the process is viewed, whether as to its simplicity, neatness, accuracy, labour, or expense, or in the efficacy and uniformity formity in purity of the product as a medicine, we do not scruple to declare, that, in comparison with any other prescription hitherto contrived, this stands without a rival. We see no reason why the Committee should have given instructions for the manipulation different from that of Mr. Hume, the inventor. To any person at all conversant in chemistry, nothing can be more perspicuous than Mr. Hume's prescription, published in the Philosophical Magazine* and in our Journal;† and, although there is room for various modifications, such as more or less dilution—adding the sulphuretted metal and nitrate at once or gradatim—the application of more or less heat, and attention to other circumstances; yet no person, we presume, would think of passing the contents of the vessel through any kind of strainer. The precipitate formed is sufficiently ponderous to subside rapidly, and to allow of its being conveniently washed off without percolation and with no great loss of time; after which, nothing further remains, the last washing being removed, than to add the supertartrate of potash and a proper quantity of water, to make a solution of tartarised antimony, by boiling these for a due time, and finishing the process in the usual way by crystallization. Some chemists assume that antimony admits of but two states of oxidation, the protoxide and peroxide: others again, particularly Berzelius, assert that there are at least four, viz. the protoxide, deutoxide, tritoxide, and tetroxide; and that the tritoxide, or third degree, according to Thenard, corresponds with the argentine flowers of antimony. We suspect, however, that water is an essential principle in some of these oxides, and that much of the intrinsic merit due to Mr. Hume's preparation may be ascribed to the precipitate being used in its *moist* state and containing hydrated oxide. We are not now prepared to enter upon the rationale of this excellent process, especially as its success is indisputable; but it is probable that the sulphuret, being exposed at once to the action of both acids, the sulphuric and nitric, of which the former is in excess, is first denuded of its sulphur by the nitric acid, when a commencement of oxidation of the antimony itself ensues; it is thus rendered receptive of the action of the sulphuric acid alone, by which the metal is eventually converted into a subsulphate, protoxide, or a mixture of both in combination with water. It is likely that the nitric acid may convert also a portion of the sulphur into sulphuric acid, so that the presence of this acid is maintained during the whole ope-

* Vol. xlv. p. 301.

† Medical and Physical Journal, June 1815.

ration.

ration. The washing of the precipitate or oxide is requisite to deprive it of the sulphate of potash; and, if it be worth while to get rid of the small portion of lime in the supertartrate of potass, a little sulphuric acid had better be added, while the solution of the emetic tartar is forming. Mr. Hume produced other prescriptions for tartarized antimony, most of them either entirely new or a peculiar modification of others: he succeeded even by that process now abandoned by the College, which has been the subject of so much petulant criticism, merely by diluting the acids; he also finds that an oxide or subsulphate of antimony, very suitable for this purpose, can be prepared, and at no great cost, by the direct application of sulphuric acid alone to the *sulphuret* of antimony. The performance of his process now received by the College is attended with no difficulty, for it admits of the apothecary making his own emetic tartar with ease, and without the aid of a chemical apparatus or furnace. Upon the whole, therefore, we sincerely concur with the College in the choice they have made, and are pleased at Dr. Powell's liberal acknowledgment of Mr. Hume's services.

*Pulvis Antimonialis.*—There can be no reasonable objection to this title, although the preparation is so much altered since its first reception as to contain but *half* the quantity of antimony. This change took place in the Pharmacopœia itself, and is again confirmed by its present edition. The translation of Pharm. Londin. of 1745, by Dr. Pemberton, commenced with a "Narrative of the Proceedings of the Committee appointed by the College of Physicians to review their Pharmacopœia," in which we find a most candid and ample display of the motives which influenced that Committee in all their transactions. We regret that we have no such guide in the present edition, otherwise we might have been furnished with the reasons for such a very great diminution of the most active material in this powder. We should have been more satisfied to have seen some remarks in Dr. Powell's note, respecting the nature of this powder and upon Dr. Pearson's analysis, published in the Philosophical Transactions, than the absurd deception quoted from the specification in the Records of Chancery. Whoever reads the prescription for *Lisle's* Powder must be convinced, that the discovery of *James's* Powder was the effect of accident; for the former consists of the same ingredients, with the exception that the hartshorn shavings are directed to be first boiled for six hours in water, then strained, dried, and powdered. Lisle directs that equal parts of this and antimony (the sulphuret) are to be set over a *moderate* fire, and stirred constantly for eight hours, or "as long as it smokes."

Now,

Now, we think the James's Powder must have been first produced by the operator while preparing Lisle's powder, fortunately neglecting his duty, by his continuing the calcination beyond this period, and, probably, to save time, placing the composition in a wind-furnace.

*Liquor Arsenicalis.*—It may be convenient to dissolve the arsenic and alkali in half the quantity of water, as the operation will be sooner finished, and consequently a smaller vessel may be employed.

*Tinctura Ferri Muriatis.*—When common rust of iron is to be procured, it will serve very well for this purpose instead of the subcarbonate. This formula gives a tincture not equal in strength to that of the P. L. of 1787; and, unless the metallic solution be submitted to some degree of heat and evaporation, there will remain an excess of acid.

*Ferri Sulphas.*—After the effervescence has ceased, the vessel with its contents, should be exposed on a sand bath, to a gentle heat, when the effervescence will recommence, and more of the metal be dissolved.

*Tinctura Ferri Ammoniata.*—This may be considered as nothing more than a very weak solution of muriated iron in proof spirit, while we already possess a strong tincture in all other respects the same, in which the spirit approaches nearly to the *rectified* strength.

*Hydrargyri Oxydum Cinereum.*—We presume that a less quantity of lime water with a very small addition of lime itself, would render the operation more convenient. The trifling quantity of lime that might remain in the product could not injure it as a remedy, for, if any did exist, it would soon become a carbonate by exposure to the air.

*Liquor Plumbi Subacetatis.*—Vinegar itself, agreeably to the original prescription of Goulard, should be used for this preparation, and not the *distilled* acid.

*Zinci Sulphas.*—Here, as in the prescription for Ferri Sulphas, it is even more necessary to apply heat after the effervescence is apparently over; this would be attended with the advantages of depriving the solution of some iron, from which the zinc is seldom or never free, and of dissolving a greater portion of the metallic base, for which purpose rather more of the zinc should be added, or till the acid be saturated.

*Oleum Sulphuratum.*—We are always glad to observe any thing like a coincidence of sentiment among the British Colleges. Here we find that our College has nearly adopted the Edinburgh formula, which is certainly an improvement.

*Sulphur Præcipitatum.*—This process of combining the sulphur with lime, and precipitating it by an acid, is not new. The acid generally employed for the purpose was the sulphuric; but the College, adopting Mr. Hume's recommendation, very properly preferred the muriatic acid.

*Potassæ Sulphuretum.*—The reduction of the quantity of the alkaline subcarbonate is quite proper, as the sulphur requires very little more, if any thing, than its own weight of the alkali.

*Gummi-Resinæ.*—We consider this as a very strange title for a class of substances in which sometimes the resinous, and in others the gummy, principle predominates. We object to it, likewise, for other reasons, for it is well known that the virtue is, in many cases, to be preserved only by keeping the original material entire. We most decidedly object to prescribing opium, whether selected or otherwise, in any other than the hard state; for the *soft* opium of to-day will certainly be somewhat harder to-morrow, and so on progressively, until it arrive spontaneously to the utmost degree of siccity; and surely it is an easy matter to add honey, syrup or mucilage, to opium, to form pills.

*Extractum Aloës Purificatum.*—Here the adjective is most essential to give precision to the title, for we are convinced that its omission has occasioned many blunders. Nevertheless, we conceive the preparation might be more specifically distinguished; for a solution of the socotorine aloes in proof spirit, or any menstruum that would separate the whole of the vegetable juice from the mere dross and contingent matters, and then inspissated, would yield what we should call an extractum aloës *purificatum*. The preparation now prescribed by the College is the mere *gum* of aloes, which, we believe, amounts to a very small portion of the imported extract. The addition to the title of purificatum, seems to have been suggested by Dr. Duncan, jun. and one of the members of the College also had proposed the word *aquosum*. There are, however, two species of aloë enumerated in the list of materia medica, and the name now chosen is unfortunately applicable to either of them.

We had made several remarks upon various other parts of this edition, but, considering the whole as a temporary production, we conceive our readers will admit, that the room already allotted is fully sufficient; we, therefore, shall hasten to conclude with a very few general observations, in the expectation that an early opportunity will enable us to undertake the more grateful task of reviewing a Pharmacopœia adopted by the whole of the British dominions.

There are many cases in which the committee might have improved without borrowing from Mr. Hume, Mr. Phillips, or any other practical chemist. We here allude to such processes cesses as the acids, neutral salts, liquor ammoniæ, many of the *metallica*, and some others where the exact chemical equivalent of one body is to be ascertained so as to fully saturate or decompose another. We are the more surprised that any error should remain on this score, since neither the committee nor Dr. Powell can be unacqainted with the *logometric scale* invented by their very ingenious colleague, Dr. Wollaston, described in the Philosophical Transactions, and which, even by Dr. Powell himself, is earnestly recommended to the attention of others.

Nothing has hitherto been more transient than the *arrangement* of a Pharmacopœia; every new edition presents a fresh scheme, a trifling variety, but no improvement. A work of this kind being chiefly employed as a book of reference, would probably be most usefully arranged by an *alphabetical* order. The most vague part of the present plan is the class of *metallica*, in which many metallic compositions are not included. Linimentum æruginis, Emplastr. Plumbi, Unguent. Hydrargyri, and even all the neutral and earthy salts, according to modern and improved theory, are metallic; potassium, sodium, and the other *new* metals are admitted as such, and their compounds are therefore metallic.

We shall conclude with pointing out two instances of the unnecessary deviation in the prescriptions of the United Kingdoms. The first is of less importance, and therefore might have been easily adjusted. On the second we shall make no remarks. The Colleges of London, Edinburgh, and Dublin, differ, without a shadow of reason, in the *time* prescribed for the maceration of the squills, for the Acetum Scillæ: the Edinburgh prescription says *seven days*, the Dublin is *four days*, and by the London Dispensatory it is only *twenty-four hours*. The tinct. veratri of the Edinburgh College is precisely the same as that of the London Dispensatory of 1745. Why, therefore, should the London College have made such an alteration in so important a medicine in their last Pharmacopœia, as to order a wine to be prepared of *less* strength than the tincture? We must deprecate these and all other discrepancies in national practice, and hope to see a single volume entitled *Pharmacopœia Universalis Britannica.*

*Reports of the Pestilential Disorder of Andalusia, which appeared at Cadiz in the Years* 1800, 1804, 1810, *and* 1813; *with a detailed Account of that fatal Epidemic as it prevailed at Gibraltar, during the Autumnal Months of* 1804: *also, Observations on the Remitting and Intermitting Fever, made in the Military Hospitals at Colchester, after the Return of the Troops from the Expedition to Zealand in* 1809. By Sir James Fellowes, M.D. Fellow of the Royal College of Physicians, &c. &c. 8vo. pp. 484. London, Longman and Co. 1815.

In our remarks on these reports, we shall confine ourselves principally to the *iter* of the diseases, so as to ascertain by collateral evidence the question concerning their contagious property. The fevers have been so repeatedly and so accurately described, that it is now well understood to be the duty of every practitioner to form his prognosis and direct his practice according to the symptoms, rather than study a name for the disease. We have heard too much of typhus, and seen too often the ill effects of bark and wine, to render it necessary any longer to descant on that subject, or to offer any other advice than that the physician should conduct himself according to existing circumstances.

In the introduction, the author, after mentioning the various scenes of service in which he has been engaged, remarks—

"It will be observed, that I have studiously avoided entering into any speculative opinions; the controversies which have sprung up of late on the question of infection and contagion, appear to have been conducted, in some instances, with an asperity of language, alike disreputable to science, and injurious to philosophical inquiry.

"After all that has been written upon this topic, we are still involved in obscurity; nor have the discoveries in chemistry satisfactorily developed the mysteries of miasmata. Future experiments may advance our knowledge of their nature; but, until they shall have been repeated with more accuracy, we are not likely to reap any advantage from discussions, which display rather the pride of science, and a disposition to singularity, than a spirit of useful research.

"It cannot, however, be doubted, that many of these writers were actuated by worthier motives, and that some, for whose abilities and acquirements I have a high respect, most firmly believe the facts they have related to confirm their own opinions, and to direct the judgment of the public in a matter on which it was so difficult to decide.

"But I feel myself in some measure particularly called upon to advert to *one* of the latest works on the subject, entitled, An Essay

Essay on the Disease called Yellow Fever, in which the ingenious author has displayed much industry and learning in endeavouring to prove that the pestilential disorder of Spain possessed no contagious quality whatever; that *its appearance at Cadiz and Gibraltar was occasioned by the morbid effects of marsh miasmata in both these places*, and that consequently any precaution founded on a belief of the importation and contagion of the disorder was objectionable, &c.

"I have occasionally noticed some of the observations of this writer, simply with a view to point out the error under which they must appear to have been made. Although I am persuaded that Dr. Bancroft would not have published them on such authorities as he has quoted in their support, had he not believed them to be true; I cannot but regret, that he had not been with me in Spain, during the prevalence of the disorder in that country, where he might have seen enough to induce him to draw very different conclusions, both from personal and local knowledge of the subject."

The first chapter comprises a topographical description of Cadiz, which contains but little new. Its confined spot, its contiguity to, and its elevation above, the sea, are all well known, as well as the extreme cleanliness in which the streets are preserved. Next follows the author's account of the origin and progress of the fever in Cadiz in 1800. After an historical enumeration of other epidemics in the same town, we are informed,

"Towards the latter end of the year 1799, the weather was remarkably severe, and it continued so during the months of January, February, March, April, and May, of the year 1800, with equal irregularity. Excessive cold, heavy rains, and violent winds, alternately succeeded each other, so that there was scarcely any appearance of spring; the heat of summer set in from the beginning of June, and by the month of August the mercury in Fahrenheit's thermometer rose to near 90 degrees, according to Gonzales's meteorological observations; and the prevalence of the east wind, or *Levante seco y abrasador*, as it is called, tended to increase the distress which the intense heat of the weather generally occasioned.

"Notwithstanding this heated atmosphere during the months of June and July, no material alteration was observed in the health of the inhabitants of Cadiz.

"About the beginning of August, the scene began to change; a certain species of fever made its appearance in the Barrio de Santa Maria, which soon attracted the notice of practitioners, from the violence and singularity of its symptoms, and from the uncommon rapidity with which its course was terminated. In this quarter of the town, described in the commencement of this work, the streets are narrower, less ventilated, and not so clean as in all the other parts, and here the poorer inhabitants, dirty in their persons, and crowded in filthy rooms, generally live together; it was amongst these subjects, already predisposed to disease, that the disorder broke out, which afterwards spread like a pestilence over a great part of Andalucia.

"The malady, although at first confined to this district, continued daily to gain ground, and in whatever house it appeared, every person belonging to the family was attacked. The frequent deaths justly alarmed the magistrates, who, anxious to check the evil, assembled together all the practitioners of the town, to deliberate on the measures of precaution necessary to be taken; but upon this as upon other occasions, where these numerous consultations have taken place, and where each individual thinks it incumbent upon him to talk a great deal, useless discussions arose, which led to nothing, and added to the general confusion and dismay. The prevailing disorder was attributed to all the causes which have ever been assigned for the production of fever, and as many names were given to it, as synochal, putrid, bilious, ephemeral, &c. &c. Those who considered it as a simple epidemic of the season, rejected all idea of contagion, and spoke only of the effects of heat, of the dry state of the atmosphere, of the exhalations from the sea, of the drains, of the low tides, and of the alteration of the bile, &c. But unhappily nothing was determined upon at this meeting, nor were any measures of precaution taken.

"In the mean time the disorder continued to make gradual progress *in the district where it broke out*, and by the middle of August the number of deaths amounted to twenty-five or thirty a day.

"The best informed persons now began seriously to inquire into the truth of the reports which were circulated, that this was a disorder of a pestilential nature, and that it had been introduced into the town; the uncertainty of some of the faculty, and the indecision of the magistrates, only tended to increase the suspicion, and added to the general alarm; it was rumoured that a vessel, called the Dolphin, had arrived from Spanish America, in which were conveyed the seeds of the disorder, and that some of the smugglers, who had been frequently on-board during her quarantine in the bay, were the first persons taken ill, in the streets of Boquete and Sopranis, in which they resided, and where it was supposed they had secreted their goods.

"The Board of Health at Cadiz was at this period merely a nominal establishment; health officers were, however, appointed, who took the duty by turns weekly, and at the time the Dolphin arrived in Cadiz harbour, Don Joseph Vallialta was the *Diputado de Sanidad*, or member of the Board of Health on duty. This person lived in the Plaza de las Tablas, near the San Juan de Dios, and not far from the Boquete, where the disease broke out. It was the general belief throughout Cadiz at the time, and it was confidently mentioned to me by many respectable inhabitants there five years afterwards, that the quarantine laws, originally weak, were on that occasion but little attended to, and that Vallialta had received received from some persons in the Dolphin a sum of money, which induced him, not only to give the ship Pratique sooner than the time required, but to connive at the communication which took place with persons from the shore, and particularly with smugglers and others, living in the Sopranis and Boquete. Two health-guards, or, more properly speaking, custom-house officers, were put on-board of the Dolphin; these were soon after taken ill; one of them survived, and the other died.

" Don José Villialta, the health-officer, who, it has been mentioned, was suspected of having connived at the smuggling which was carrying on from the Dolphin, was reported to have caught the disease, and, it was added, that, struck with remorse at the dreadful effects which he foresaw were likely to result from his misconduct, and feeling deeply the reproaches which were heaped upon him by his acquaintance, he gave himself up to despondency, and died in a few days of the prevailing fever, about the 20th.

" The circumstance of Villialta's illness, and the exact period of his death, are faithfully recorded at Cadiz: the event confirmed the general opinion at the time, and occasioned so much alarm amongst the inhabitants of that quarter of the town (Barrio de Santa Maria), that on the 23d a great crowd of persons assembled before the house of Don Francisco Marti, Syndico Personero, or head of the municipality, and supplicated him in the most earnest manner to permit them to join in procession, and to carry out the image of our saviour (Nuestro Padre Jesus) from the church of Santa Maria.

" Such was the terror of this fanatic people, that they conceived themselves the objects of offended Heaven, and imagined that by following the cross with humility, they should effectually appease the anger of the Deity. The magistrate, dreading this assemblage of persons in a part of the town where the disorder was spreading, in vain endeavoured to quiet their apprehensions, but all reasoning was ineffectual, and the procession took place, passing through the Sopranis and Boquete, and from thence to other quarters of the city, where the disorder had not yet appeared; but, in five days after, cases of the fever were reported in the other Barrios, and, on the 28th of August, there were 157 deaths in Cadiz.

" It was now ordered that the dead should be conveyed away in carts, and buried outside the town; the ringing of bells was prohibited, and every measure was adopted to tranquillize the minds of the people; but the dread of this great calamity was so strongly impressed on every individual, that it only increased the aptitude to take the disease, and many instances occurred of deaths accelerated solely by the terror thus induced.

" The spare diet, and an abuse of preservative means, brought on such debility as to be fatal to many persons; and to such a degree did the confidence in preventatives prevail, that scarcely a person was to be seen without a handkerchief steeped in thieves' vinegar; others kept garlic constantly in their mouths, in their bosoms, bosoms, and in their pockets; and others, again, wore aromatic and cordial amulets: this practice greatly contributed to affect the nervous system, and to increase the predisposition to the disease.

"Cadiz was now become a desolate and melancholy place; and by the middle of the month of September, the deaths amounted daily to 200. At this period, the air, from its stagnant state, became so vitiated, that its noxious qualities affected even animals: canary-birds died with the blood issuing from their bills; and in all the neighbouring towns which were afterwards infected, no sparrow ever appeared during the epidemic. In fact, the disorder became general; whole families, at once under its influence, remained without comfort or assistance; the hospitals were filled, and no one could be found to attend upon the sick; the apothecaries' shops were shut; the greater part of the practitioners in the town were themselves taken ill, and many of them were victims of this terrible scourge.

"Although several of the leading members and magistrates of the city had been early carried off by the fever, the most perfect order prevailed in Cadiz, and none of those irregularities and disgraceful scenes took place which had occurred in other towns during the period of public calamity.

"The mortality, which was great in the beginning, began to diminish on the approach of autumn. Early in October, the British fleet, under Lord Keith, appeared before Cadiz; and this novel sight produced an extraordinary effect upon the minds of the people. Thus the fear of an attack roused them to individual exertions for their defence; and the inhabitants, who before had given themselves up to despondency, and thought only of their domestic misfortunes, now left their close infected houses, and by *respiring a purer air*, contributed essentially to their own recovery. Whatever may have been the cause, whether proceeding from the new impressions which were excited by the activity of the scene, or by the efforts which all were called out to make, to resist an expected landing of the British troops, or from the change of the season, certain it is, that the public health was so perceptibly improved, that there were scarcely any deaths reported towards the end of the month. The contagion was, however, conveyed, by emigrants from Cadiz, to the neighbouring towns; the communication had been cut off too late, the disorder spread rapidly to them all; and the attempts to check its progress afterwards were ineffectual.

"Those who had fled from Cadiz, now endeavoured to return; and, although the gates were ordered to be closed against them, yet many found means to get in, a great part of whom were shortly after attacked with the disease, and fell victims to their indiscretion. Thus it was clearly proved that the poisonous *miasmata* were still active, and that the disorder acquired fresh vigour in proportion as new subjects presented themselves to its influence.

"Various means were devised to purify the air of the town, and and to fumigate the public buildings, &c. Recourse was had to the firing of cannon, (a dangerous and erroneous practice); and at length, about the 12th of November, the city was declared in a state of health by the celebration of *Te Deum.*

"The number of persons attacked with this disease in Cadiz, from the beginning of August to the first week in November 1800, amounted to 48,688; of those who recovered and were convalescent, 40,699; and of those who died within the city, to 7,292. In Seville the mortality exceeded 22,000, and in Xeres the number of deaths was returned at 10,000."

A note is added to shew the mistake of Dr. Bancroft, who imputes the disease to marsh miasmata, which do not exist near Cadiz.

An account of the disease, with its symptoms, follows, in which we have no reason to suspect the slightest inaccuracy; but the account can only interest those who are likely to visit the same scene; and for these any thing less than the whole description would be unsatisfactory. Suffice it to say, that the general history is very similar to what we meet in tropical fevers.

The second report is on the epidemic at Gibraltar, and commences with a description of that celebrated rock. As this is now pretty generally known, we shall only remark, that the neutral ground, though low, is sandy, and not productive of marsh miasmata. Concerning the origin and progress of the disease, we extract the following remarks:

"On the 27th of August, 1804, the intelligence from Malaga continuing to be more and more alarming, Sir Thomas Trigge issued a proclamation, prohibiting all vessels coming from the eastward or westward of Malaga from entering the harbour, and admitting to pratique those only from the eastward of Carthagena and westward of Tarifa.

"On the 28th, the day following, it was reported that a person of the name of *Santos* had arrived in the garrison from Cadiz, where the fever then prevailed, and that he and several of his family were taken ill. Little attention seems to have been paid to these rumours until a few days afterwards, when the assistant-surgeon of the royal artillery, Mr. Kenning, reported to Dr. Nooth, as chief of the medical department in the garrison, that he had some married people belonging to the corps under his care, whose complaints had assumed an appearance which embarrassed and alarmed him; on examination of these patients, it was declared that they laboured under the bilious remittent fever common to warm climates, and directions were accordingly given for its treatment. The facts that were afterwards ascertained respecting the arrival of Santos from Cadiz, and his being the person who introduced the infection into the garrison are upon record, and they carry a strong conviction of the probable mode in which he had contracted the disease; indeed, from his own confession, and the oath of a respectable witness, it appeared that he had left an infected house at Cadiz, that he had been three times in company with a person actually labouring under disease, about the 23d or 24th of August, that he arrived at Gibraltar the 25th, was taken ill the 26th, and was seen by the French practitioner, Monsieur Jay, on the 27th of August; and that in less than eight days after his being attacked, his mother, two aunts, one brother, and two sisters, all residing in the house, were also seized with a disorder of a similar nature, and of which *no case* was known to have occurred in the garrison previous to young Santos being taken ill. The proofs, therefore, that young Santos was the first person attacked with the disease, and that he had been the means of introducing it into the garrison, appear to be as strong and conclusive as the circumstances of the case could possibly admit of, especially when it is considered how the malady spread from the house of Santos to the adjoining buildings, whilst the rest of the garrison were totally exempt from it.

"For several days the disorder was confined to the range of buildings to which it had been traced, and where Santos lived, and it was observed to make a gradual progress amongst the different families who resided there, and to spread to the sheds in the neighbourhood.

"This peculiarity, added to the rapidity with which, in some instances, the fever terminated its course, excited very general alarm.

"But the death of a Bombadier of the Royal Artillery, and his wife, after a few days illness, on the 12th of September, confirmed the suspicion of there being an unusually fatal disorder in the garrison, and excited the attention of the officers of the corps, especially of Captain (now Lieutenant-Colonel) Wright, to whose company those persons belonged: he was indefatigable in endeavouring to ascertain every fact upon the subject, and, with the secretary of the garrison, Captain (now Major) Dodd, proceeded to the examination of the French practitioner Monsieur Jay, who attended all the first cases of the fever amongst the inhabitants, and Assistant-surgeon Kenning, and others who could give any information. It appeared to them from their description of the prevailing disease, that it resembled the account given of the epidemic of Cadiz in 1800, by some of the Spanish authors, whose works Col. Wright and Major Dodd had read, so that no doubt existed in their minds as to the nature of the malady. Nothing was, however, decided upon until the 15th of September, when the Spanish priest, Hoyera, a man of respectability and of great authority amongst the Catholics in Gibraltar, called at the secretary's office early in the morning, and informed Captain Dodd, that *he had been to visit some sick inhabitants, and that he had good reason to believe that a very malignant and fatal disorder* then

*then prevailed in the garrison; that it appeared the same as that which he had seen near Seville in* 1800, and he conjured the secretary, in the most solemn manner, to acquaint the lieutenant-governor of his declaration."

We shall not pursue the account any further than to remark, that the disease increased till November, during which month it ceased; that the inhabitants were principally affected, though the garrison suffered also; that in the lazaretto the mortality, as usual, was very great; that those who had passed through the yellow fever in America escaped; that most of the attendants on the sick died.

As our opinion differs from the author's, we conceive it our duty to add the following report from the appendix.

"*Gibraltar, 9th March,* 1805.

"Mr. Pratt, Master Cooper in the Naval Victualling Yard at Gibraltar, deposes, that he left this garrison about the 30th July, 1804, with a passport from General Sir Thomas Trigge, with an intention of going to Cadiz. He went in a boat to Algeciras, and from thence by land. He arrived at Cadiz the 3d or 4th of August. He did not understand that the town was unusually unhealthy. He lodged at the tavern Del Sol, in the street Hondillo, where he remained about fifteen or sixteen days, when he was taken ill; having continued so for eight days, he had symptoms of black or bloody vomiting; at this he was much alarmed, and, fearing lest he should be sent to the public hospital, he removed to another quarter of the town, and ultimately recovered. He had, however, a very yellow look, which prevented the master of a vessel from taking him on board and bringing him to Gibraltar, lest it might be the means of putting the vessel in quarantine.

"Mr. Pratt further deposes, that he and the Captain of his Privateer went into a barber's shop to be shaved and to get their hair combed; that shortly after they both complained of pains in the head, and they were immediately after taken ill. The Captain of the Privateer lived in the same house with him, but was removed to the hospital, where he died. He also deposes, that a man belonging to Gibraltar, named *Santos,* who was the first person attacked with the fever here, lived many days in the same tavern with him and the Captain of the Privateer; that *Santos* was in the same room with him (Pratt) whilst he was ill of a fever, which, however, he endeavoured to conceal, for fear (as already stated) of being compelled to go to the hospital; that *Santos* returned hither in the vessel in which he (Pratt) had been refused a passage; and that whilst he was in the tavern, several persons were ill. He was told that the disorder generally attacked strangers, and was fatal to them. That he was attended when he removed from the tavern by a man, to whom he afterwards gave some of his clothes; and that this man was, shortly after receiving them, taken ill, and died.

 "I swear

"I swear to the foregoing statement being true, to the best of my memory and recollection.

(*Signed*) JOSEPH PRAT

*Witness*, THOMAS DODD.

Sworn before me this 4th day of March, 1805.

(*Signed*) H. E. FOX."

A chapter follows on the history and treatment of the fever. For the reasons before-mentioned, we should pass this entirely over. It contains, indeed, some remarks on the progress of the epidemic, but they are not sufficiently explicit, on account of the general terror which seemed to prevail.

The succeeding chapter is on the pestilential fever at Malaga in 1803 and 1804. These particulars were collected by the author in 1806. Here again we are referred, as in most other instances of epidemics in sea-ports, to the nefarious practices of a noted smuggler. On this subject we shall say more on a future occasion: at present we shall only remark, that it was a *post orbit* report of a dying man's confession. As is generally the case, as if by the kindness of Providence to give notice to the inhabitants of a pestilential town, the disease did not spread for a period of thirty-five or thirty-six days after. This is the more remarkable, because the man smuggled tobacco and cotton, articles very likely to be circulated. The family of the deceased seem to have been the wisest of any: they not only left the house but the town, and the house remained empty when the report was made a twelvemonth afterwards. But another smuggler, of a notoriously bad character, "likely to do any thing for gain," secreted a person in his house, who died, and was buried privately.

The following passage is a general summary of the question.

"'*The separating of the sick* (says Arejula) *from those in health, and avoiding all communication between one and another, is the only measure which, in such cases, can be depended upon to prevent the propagation of the malady.* These observations explain why a proportionably greater number perish in hospitals than in houses, even allowing the sick to be equally well attended in both; and, if it were possible to convince people that the patients should be separated, in their first attack, in order to cure them, many more would be saved, and the disorder would not become epidemic.'

"I am satisfied that much of the mortality in Spain has arisen from the total want of attention to this judicious remark of Arejula. There can be no doubt that this fever would, by proper management, become less formidable; and that there is scarcely a disease disease known, however infectious, which may not become milder, or lose its infecting power by early attention to *separation, cleanliness,* or *ventilation.*"

It is further observed, that the felons confined in the *presidio*, being kept clean, in a well-ventilated house, and free from every communication, all escaped.

On this occasion we shall remark, that it is at least a doubtful inference from the above whether the mortality in hospitals does not arise from the crowding of the sick, and not from the mere intercourse of the healthy with the sick. The observation that all infectious diseases become milder, and lose their infectious property, by these cautions, is not sufficiently explained, since no ventilation or cleanliness lessens the infectious property of small-pox, or of the other exanthemata.

The third report is on the epidemic at Cadiz in 1810. This is interesting, particularly as it contains some remarks on Dr. Bancroft, by which we should suspect that gentleman of precipitation in giving his reports. We shall extract some of those passages which relate to the question of contagion.

"On the 11th of September, 1810, the physician to the Board of Health first discovered some persons labouring under symptoms of fever, similar to what he had observed in 1800 and 1804; and it appeared from the reports which he made to the Junta (which were not communicated to me until some time afterwards), that the disorder was contagious, having spread gradually on the quarter of the town where it broke out, four out of five of the first family attacked having died, and particularly as the only individual who survived was *not* ill, *having passed through the disease in the epidemic of* 1800. The greatest secrecy was, however, observed, and nothing was known of the existence of any fever in the town except to the Board of Health, and the Government, until the beginning of October.

"Reports were now circulated, that there was a fever of a *suspicious* nature in Cadiz, but they were formally contradicted by those in authority (which has always been the mistaken policy in Spain), and no one dared to state publicly the fact. I considered it, however, to be my duty to communicate the information I had received to General Graham, and in consequence of my recommendation to cut off as much as possible the communication with the town and the troops at the Isla de Leon, a general order was issued the day following, prohibiting any officer or soldier from going into Cadiz, except on the most urgent public service. At the same time a communication was made to Sir Richard Keates, of the measures that were adopted to preserve the health of the army; and it was recommended to him to confine the officers and crews as much as possible to their ships, and to interdict all unnecessary intercourse with the shore.

 "These

"These precautions were taken on the part of the British general and admiral previous to any public notification of the existence of disease by the Spanish government.

"On the 16th of October, I made a report to General Graham, acquainting him with the opinion of professor Arejula, and of the principal physicians in Cadiz, upon whom I called officially for information, respecting the actual state of the public health. They all agreed in strongly recommending the Board of Health to redouble their vigilance, and to enforce the regulations published in the Edict, to prevent the sick from being crowded in the public hospitals, and to promote the cleanliness and ventilation of the houses, &c. &c. Their opinion was, however, worded in a very cautious manner, but, as it was the first official declaration that was made on the subject, it was sufficient to justify the advice which had been given to the General in command; in fact, the disorder gradually increased, and it afterwards appeared in several parts of the town. The number of deaths from the beginning of September to the 24th of October, were now known to have amounted to 929: the population was then estimated at 120,000 persons, without including the numerous families who lived on-board of vessels in the bay.

"*No case* of the prevailing disorder had at this time been observed in any of the military hospitals, nor had an instance of contagious fever been known amongst the troops. In my report to General Graham of the 16th October, it was remarked that the ordinary complaints of the soldiers were then very few, and remarkably mild for the season, and that within the last two months they had considerably diminished.

"This exemption of disease amongst the British troops was a matter of astonishment to the Junta of health, particularly as it was a well-known fact that the prevailing sickness was principally confined at this period to strangers and to persons from northern climates, and even to the Spaniards who had fled to Cadiz from the northern provinces of Spain, and to whom the disorder was most fatal."

After this, however, we find that the disease appeared in the English barracks within Cadiz; that Dr. Snow very judiciously advised removing the soldiers from their quarters, or, if that could not be accomplished, that they should have more space for each, but circumstances did not permit either.

"The pestilential disorder (we are informed) continued to prevail throughout the month of October, and it was confined principally to the inhabitants who had not previously been attacked with it in 1800 and 1804, and to strangers recently arrived in Cadiz. *No case of the fever* appeared amongst the British troops at the Isla de Leon (which town, it has been observed, was surrounded by marshes and salt-pans. But it has been shewn that the disease had broken out in the barracks in Cadiz, where some of of our corps were quartered, and, although it had attacked a considerable number of the men, before we were enabled to check its progress, our loss altogether did not amount to more than *twenty-five* men, including two officers and a clerk in the commissariat department.

"Towards the beginning of November, the disorder appeared to be upon the decline, for it manifested a tendency to terminate by assuming, in several instances, a variety of forms and modifications in the symptoms, which preceded its final cessation, about the end of the month.

"During this epidemic, between two or three thousand inhabitants were carried off by the fever; but the number of deaths in Cadiz, according to the yearly return for 1810, amounted to 4305. Dr. Mellado, physician to the Board of Health, published an account of this fever, entitled 'Historia de la Epidemia padecida en Cadiz el ano de 1810.' The ingenious author has proved its contagious nature by numerous well-attested facts; and he has endeavoured to shew, from official documents, that it did not originate in Cadiz. But he candidly acknowledges the difficulty of tracing it to its source, and of ascertaining who were the first individuals attacked. It was a very remarkable circumstance, that the pestilential fever should have broken out in one of the Canary Islands, where such a disorder had never appeared before."

It is not less remarkable that at the Canaries also no discovery could me made as to the means of its introduction. The remarks on Dr. Bancroft only shew the haste with which that physician compiled this part of his work.

The fourth report contains an account of the epidemic at Cadiz in the year 1813. This commences with the following account of a typhus fever, and the judicious manner in which its influence was stopped.

"After the termination of the epidemic in December 1810, there was no appearance of a return of it until 1813; but towards the latter end of January 1811, two English transports (Metcalf and Phylleria) arrived in the bay of Cadiz, from Gibraltar, having between 4 and 500 German recruits on board, most of whom had been selected to form a German battalion, from the deserters of the French army, and from the prisons and hospitals in Carthagena.

"These Germans had been kept on board under quarantine for upwards of a month, in Gibraltar bay; and unfortunately on the arrival of the transport at Cadiz, the weather became so tempestuous, that the crews of those vessels and the soldiers were obliged to keep below. When the weather moderated, every assistance was afforded them; but it proved that, during *the few days* that the hatches were covered over in consequence of the heavy rains, a complete typhus fever had been formed; that the men (who appeared to be well whilst they had been kept upon deck constantly, and the fresh air had been suffered to pass through the ship) were falling down with the symptoms of a malignant disorder, the germs of which it was evident had been brought by them from the French army, or from the jails and hospitals of Carthagena, and had exploded into fever in the vitiated air by which they were surrounded, in the close and crowded 'tween-decks.

"Under these circumstances, the most prompt and decisive measures became absolutely necessary. Nearly 100 of the Germans, and several of the crew, were found labouring under fever; and the most serious apprehensions were entertained of the disorder spreading amongst the different transports in the bay. The first object was to separate the remainder of the men on-board who appeared in health. The difficulty, however, of landing any of them was great, as the Spaniards were extremely fearful of the recurrence of the disease of the preceding year, and it was the wish of the General in command to preclude all possibility of any misconstruction on the part of the Spanish government, in the adoption of such a measure as landing the sick, however urgent and necessary.

"But the situation I held as member of the Board of Health, afforded me the sanction of the government to take upon my own responsibility the steps that were pursued.

"The whole of the men were directed to be washed in warm soap and water on-board of the transports, under the inspection of medical officers, and their dirty infected clothing to be thrown overboard; each man was supplied with fresh hospital dresses on being put into the boats, and 400 of them were in this state landed and sent to a temporary hospital, a mile from the town.

"Nearly 100 of the sick remained on-board of one transport, whilst the one which had been evacuated was thoroughly cleansed, ventilated, and fumigated with sulphur, after which they were removed into it.

"Thus, in the course of a few days, the effects of this process, with the change of diet, &c. were evident amongst the sick."

It is impossible not to approve of all these measures, but it is equally fair to remark, that the disease occurred in spring, and at a temperature never sufficient for the existence of yellow fever.

Though Cadiz, from the unsettled state of Spain, had been very much crowded, yet no particular mortality occurred during the years 1811 and 1812. Till the autumn of 1813, only the common diseases incident to other towns prevailed; but in the autumn the yellow fever again prevailed with all the violence that it could well assume, considering the number of people who left the city with the government, and the numbers who had passed through the disease during the two last epidemics; not one of whom was affected. But we do not find that the author attempts to have discovered any means by which the fever was introduced. The few instances which occurred at Gibraltar do not not appear to have been more than what are commonly met with at that season of the year. As is usual, of the new comers, scarcely any escaped. Some remarks follow on the different modes of treating the fever, on which we do not think ourselves authorised to give an opinion, till we see all that the Spanish physicians have to urge in their own defence. It is also very justly observed by Sir J. F. that much depends on the period of the fever, and that the British physicians seldom saw their patients before the disease was completely formed. The mode of life, also, among the British troops, may have produced a considerable difference in the form of their acute diseases.

A long report follows on the bilious or autumnal remitting and intermitting fever (called the Walcheren fever), with remarks on the treatment of that disease, as it appeared in the military hospitals at Colchester in the months of September, October, November, and December, 1809. On this subject we have already so many pages in our Journal, as to render it unnecessary to enter into any particular details.

Some observations follow by way of conclusion, of which we shall extract enough to shew how inconclusive most of our inferences are.

"Speculations on the origin and nature of the pestilential disorder may be interesting and useful at seasons, and in places remote from its influence; but when the danger is imminent, they too often tend to embarrass and distract the practitioner, and retard the adoption of those simple measures of precaution, of which the success is the more complete in proportion to the promptness with which they are applied. To these means, popular custom and national prejudice are formidable obstacles of themselves, and, if strengthened by scientific objections, they acquire additional force, and are in consequence doubly mischievous. It has been shewn that this fatality has invariably attended the discussions of the Spanish authorities on the breaking out of each epidemic, and that notwithstanding the frequency of this calamity, no advantage appears to have been derived from experience.

"The truth of this observation is strongly exemplified in the report of the year 1813, where it has been stated that the arguments of the orator Mexia, in his speech to the Cortes, occasioned a most delusive calm; for the people, ever too prone to embrace a doctrine soothing to their indolence, and assuasive of their fears, remained satisfied under the assurance that the disorder, which was beginning to prevail in the town, was not of a contagious nature, and that they were consequently exempted from any troublesome measures of precaution.

"How many victims might have been spared, if the salutary lessons of experience had been attended to in the first instance, and if a wise and vigilant apprehension had taken place of that pernicious cious security which was so soon to subside into general panic and alarm!

"Although every temptation to indulge in speculative opinions has been avoided in these reports, yet the inquiries and results which they contain will, it is hoped, be of some service to the future investigation of this interesting subject.

"It may, however, be proper to add a few remarks respecting the contagious nature of the disorder, its peculiarities, the general mode of treatment, and the means most applicable for its prevention.

"The terms contagion and infection have had a meaning far more extensive than precise, and they have been variously applied and distinguished; the most satisfactory discrimination on the subject is to be found in the Second Report of the Board of Health in London to the Privy Council, which, as it accords with my views of the question of the utility of mineral acid gasses in destroying infection, I shall cite in this place. 'Such is the subtle nature of all the poisons generated in animal bodies, that we are totally ignorant upon what their properties and powers depend, and cannot, upon general principles, apply to one of these poisons, what we have learned of another. We are led to make these observations, because the language commonly used on this subject may lead to error. Contagion and infection are supposed to be applied to sound persons; but, by a common figure in language, they are often used to express the poison itself, and such poisons go by the name of contagious and infectious; and what destroys contagion in one case, it has been inferred, would destroy it in another; but there is no foundation for this broad conclusion; for example's sake, we will suppose it proved that the acid of nitre, or of common salt, would destroy the poison of the gaol-fever, we cannot from thence infer that it would destroy that of the measles, small-pox, or the plague;' nor, I would add, of the pestilential fever of Spain.

"The observations made in that country, during the later epidemics, prove, beyond all doubt, the inefficacy of the boasted virtues of the mineral acid gasses. The completest trial was made of them by the Professor of Chemistry, Dr. Arejula, after the termination of the fever in 1800. He wrote a dissertation on the subject of the acid fumigations, which was published by order of the Spanish government; and his plan (borrowed, as he asserts, from him by Guyton Morveau) was afterwards generally adopted in most of the principal towns of Andalusia.

"In 1803 the pestilential fever (as it has been shewn) broke out again in Malaga, and not in Cadiz, as some have pretended; and Arejula endeavoured to ascertain, by the most careful and accurate experiments, how far the acid gases were effectual in preventing the spreading of the infectious miasms.

"Two physicians of the Spanish navy, Don Mateo Perez, and Don José Maria Salamanca, assisted him on these several occasions, but, after the most careful observation they did not perceive any difference difference between the houses which were all day filled with the fumes of the acid gases, and those in which they had not been employed; for the disorder prevailed with equal fatality in both, and attacked individuals in the different buildings whether they were fumigated or not."

On the above extract we wish to ask our readers what are the satisfactory discriminations marked by the Board of Health, and which accord so well with Sir James's opinion. That there is much caution we admit, and a suggestion that common language is incorrect, and also that there may be a difference in different contagions or infections; but sure there can be no great novelty in these remarks.

---

### *Edinburgh Medical and Surgical Journal*, No. XLIV. for October, 1815.

(Continued from p. 431.)

### Art. VII.—*Letter from* Mr. Carmichael *in reply to the Review of his Essay, &c.*

The insertion of this letter is a sufficient apology for all the sins of which we before felt disposed to accuse our brother journalists, had it been consistent with propriety for us to notice this part of their labours. Valuable as the whole paper is, we shall extract only those parts, the insertion of which we conceive particularly to mark the candour of the managers of the Journal, because they relate to *words* and *language*, very tender subjects with one class of writers.

"After this instance of candour, (says Mr. C.) he [the Reviewer] proceeds to urge his opinion, in opposition to mine, that these phagedenic and sloughing ulcers are 'truly syphilitic in the outset; but that the syphilitic virus had been *eradicated by the specific*,*

---

"* If an author might presume to offer advice to a reviewer, I would strongly recommend our critic to peruse a page or two of Dr. Adams on the subject of this very phraseology, which, if it were less absurd and unphilosophic than Adams conceives, a gentleman in his vocation ought surely, before this time of day, to have abandoned.—See Adams on Morbid Poison, 2d edit. from p. 147 to 151."

On the perusal of this note, we could not help recollecting the appendage to this work, which we usually meet with in Callow's Catalogues:—" This volume is valuable in another point of view, because it inculcates the habit of analysing diseases, and shews the importance of minute attention in tracing the history and progress of every series of morbid action. Vide Edinburgh Journal, vol. iii." —As there seems such a coincidence of sentiment between Mr. Carmichael and his reviewer, we cannot doubt the attention of the latter to the advice given by the former.

and these phagedenic dispositions had ensued, either as the sequelæ of lues venerea, or the effects of mercury in a bad habit of body, to borrow the words of an author very well informed upon this subject. For we are disposed to believe, with this able and experienced writer, (Mr. John Pearson, in his admirable work on the Effects of various Articles of the Materia Medica in Lues Venerea,) that the venereal virus, when introduced into the system, often gives rise to morbid appearances, which do not in any proper sense partake of the nature of the remote cause.'

"Now, as I have given several instances of primary ulcers, which were phagedenic or sloughing, and as I stated that I had an opportunity of observing from the outset, that they had not any of the characters of chancre, I cannot well conjecture how, in contradiction of those facts, the reviewer can theorize these ulcers into sequelæ of syphilis, which, in plain English, I believe, means the sequel or consequences of lues venerea, or, as the vulgar call it, *the dregs* of the disorder. And in opposition to the fact, that many of these patients were cured without mercury, (see particularly Cases LI. LII. LVII. and LXI.): still less can I comprehend why the critic adopts the still more inconsistent hypothesis that these ulcers were the effects of mercury on a bad habit of body."

The following extract is directed to the same object.

"The reviewer continues: 'We confess that we are a little surprised to find the author setting out with this annunciation, that the task will not be very burthensome, for one species *of eruption* only remains to be discussed, all the others which I have met with having been already described and traced to their source (p. 187). We began to recollect the extreme variety of cutaneous eruptions which we imagined our own observation had presented to us of equivocal character, and accompanied by nodes and pains of the limbs, by ulcerations of the throat, inflammations of the eyes, emaciation, &c.' The reviewer here thinks proper not to understand the meaning I applied to the term '*species*.' All the venereal eruptions I had met with were either scaly, papular, pustular, or tubercular. Those of the three first characters were already decribed; the last alone remained to be considered. This, from the context, was so evidently my meaning, that it could only be wilfully mistaken. Our critic, however, possibly adopted his meaning of the passage, for the purpose of contrasting it with the more prudent caution of a gentleman for whom I, in common with the public at large, have the highest respect; but Mr. Pearson can derive but little pride from the adulation of an eulogist who proves, when he criticises, that he feels not what he most ought to feel, a passion for truth. The encomiastic strain thus proceeds: 'We remembered the difficulties expressed by one of the most learned and able surgeons of the age, already quoted, after an attendance of thirty years on the Lock Hospital of London, and the enjoyment of extensive general practice, respecting these spurious appearances. 'I have not yet attained to that complete and satisfactory knowledge of the *cachexia syphiloidea* which would authorize me to obtrude a publication on the subject; but the experience I have already had in the treatment of that *multiform disease*,' &c. &c.

"Now, as I am fully of opinion, and that opinion strongly built upon facts, that those spurious appearances, as they are termed by the reviewer, are not the product of the syphilitic, but of other poisons, I ought to have been pardoned for not adopting the opinion of Mr. Pearson, however in general I value it, nor the term he employs of *cachexia syphiloidea*, which is so highly admired by his eulogist, and which, I admit, was a very good appellation in the state of our knowledge when Mr. Pearson first used it; but, as we were then ignorant of the subject, he was necessarily compelled to resort to the common shift, of choosing a term calculated to satisfy our ignorance when real knowledge is wanting."

The author proceeds afterwards to vindicate his attempt at superseding one morbid poison by another; on the authority of Mr. Hunter and some other writers. We conceive his success might have proved his apology.

We would offer more extracts from this paper, but many of the arguments are already anticipated in our review of Mr. C.'s work. It is, however, with much pleasure that we dwell on the opportunity an author is thus allowed of vindicating himself. His paper is fairly given to the reader, without the mutilation of extracts, and without the artful suggestions or interruptions of the journalist. How different this from the attempts made a few years ago, by a junto of writers, who, under the pretence of a medical review, condemned without understanding, or misrepresented from design, whatever came not from themselves or their party; and how much to the honour of the medical public, that, in spite of every attempt, and even of some good writing, so undeserving a work should be so short-lived. No stronger proof can be desired of the advantage the public derives from a periodical work, which, by admitting original communications as well as reviews, affords every author an opportunity of defending himself. When we see with what candour Mr. Carmichael is treated, we are constrained to believe what we have often been told, that the gall sprinkled on him and some other writers has been fermented in a more southern meridian. We shall conclude with advising our northern friends, in their future reports—*Cavete a fermento*, &c.

Art. VIII.—*Cases of Re-production and Re-union of part of the Finger after Accidents; and Case of Absorption of the Water in Hydrocele from mental Agitation.* By Edward Baynes Fletcher, Surgeon.

Not being acquainted with the author of this paper, we shall only remark, as his case occurred nearly five years ago, we cannot help doubting the correctness of the writer's memory. We recollect of no other instance of the kind excepting a solitary one in the Edinburgh Medical Essays, in which the glans penis was renewed. The latter is, however, a part extremely sanguiferous, and of very uncertain magnitude, so that a conical fungus might, when skinned over, have gradually assumed such a form; the part itself is also less complicated, having no nail, like the end of the finger. The subject too was, we believe, very young. What the age of the following was, we are not informed.

"On the 24th of September, 1810, (we read,) Mr. Hedley, carrier between Morpeth and Newcastle-upon-Tyne, I was then practising in Morpeth, came to my shop to get his finger dressed, the end of which was a few minutes before crushed off by a waggon wheel. On examination, I found the end of the finger, from the third joint to the point, including the nail, entirely cut off in an oblique direction, and hanging by a small portion of the cuticle. There was no injury of the phalanx. Not having sufficient confidence in the powers of nature to re-unite the parts, I did what the surgeon in the blacksmith's case did,* divided the skin with my scissars, and contented myself with dressing the stump. Nature, however, was kinder to him than I was; for, during the process of the cure, she restored to him what I, in want of confidence in her power, had deprived him of. Granulations soon began to form, and fill up the wounded surface; and, though the process was slow, the finger recovered its original shape and appearance, but wanting a nail, which, however, in a few weeks, made its ground good. The lesson placed before me in this case, determined me, on any similar occurrence, to attempt to shorten the process, by endeavours at re-union,—a practice I had occasion to put into force last summer."

To the other cases we can make no objection, especially if the foregoing should be confirmed.

Art. IX.—*Case of Re-union of the Thumb, communicated in a Letter to Dr. William Balfour, Edinburgh.* By Thomas Hunter, Esq. Surgeon, Port Glasgow.

In order to authenticate this case, Mr. Hunter has thought it right to procure the signature of the patient and his employer; but, interesting as it is, and creditable to the author, the whole wonder ceases after reading the former article.

Art. X.—*Case of Chronic Hydrocephalus, in which the Mental Faculties remained unimpaired till a short time before Death.* By Dr. Reed, Kilmarnock.

Such cases are well worth recording; but we hope, after

"* Vide note to Dr. Balfour's Case, page 425, vol. x."

the the remarks of Dr. Spurzheim, confirmed by Sir Everard Home, that the appearance of the brain will in future be more minutely described, in order to satisfy the world concerning the justness of the cause to which Spurzheim ascribes the retention of their senses in such subjects.

Art. XI.—*Case of a Child born between the Fourth and Fifth Month, and brought up.* By JOHN RODMAN, M.D. Paisley.

This is a truly interesting history. The author imputes his success principally to preserving the child's temperature by animal heat: whenever artificial heat was substituted, the child suffered; or if the heat was increased beyond the human temperature, it shewed evident marks of distress. The case does great honour to Dr. Rodman and his coadjutors.

Art. XII.—*Remarks on the Treatment of Epilepsy, and some other Nervous Diseases.* By J. C. PRICHARD, M.D.

This is a sensible paper, but the writer takes only a partial, or, as we veterans should call it, a recent view of medicine, when he imputes to Dr. Hamilton's book the removal of prejudices concerning the treatment of what are called nervous complaints. It is very true that the influence of the Cullenian doctrine, and, perhaps, of the Brunonian system, had been very extensive, and we are glad to find so popular a work as Dr. Hamilton's has been the means of superseding it.

We shall give a brief extract of the cases, and hope, if possible, to learn, in a future number, the sequel of each.

The first occurred in December, 1811. The subject was

"A sailor boy, about 15 years of age, of slender make, was seized, four days ago, with a fit while on board ship. He felt at first a peculiar sensation, attended with convulsive motion in one of his hands, which presently rose along the arm to his head. When it had reached his head, he instantly fell down senseless, and all his limbs were convulsed. He foamed at the mouth. After the fit went off, he remained stupid, and affected with violent pain in his head. This morning he had another fit, which came on in the same manner, and was similar in every respect. Pulse 100, weak and small. The bowels had been constipated, but he has taken some laxative medicine since the first fit.

℞. Pulv. jalap. gr. xii.

Submuriat. hydrarg. gr. iv.

Ol. caryoph. gtt. i. M. ft.

Pulv. sumendus statim, et repetendus secundo quoque die.

"December 13th.—Since he began to take the medicines, he has twice been alarmed by the previous symptoms of the fit. The sensation and convulsive motion in the hand came on as before, but ascended no higher than the fore-arm. No affection of the head or or other symptom followed. He feels himself much better, but still complains of head-ach.

Repet. pulv. et singuli grana Jalapii xvi. contineant.

"December 20th.—Has had no fit or previous symptoms. Is free from head-ach, and well in other respects. Let him continue the powders.

"He continued some time to take the medicines, and had no return or threatening of his complaint until the autumn of the following year, when he came to me and told me that he had just had a fit. His bowels had been for some time constipated. I ordered him to take a cathartic powder every day, and he had no return of the convulsion. I have reason to suppose that he has suffered no attack since that time.

"A curious circumstance in this case was, the sensation and convulsive motion in the hand which preceded the attack, and which may be considered as an example of the Aura epileptica. The Aura epileptica has been supposed to point out the seat of a local irritation, which excites the fit; and it has been proposed, accordingly, to divide the nerves in their course from the point where it arises, towards the head. But in this case, if there was any local irritation exciting the convulsions, it appears to have been situated in the intestinal canal; and the disease was removed by remedies which had no reference to the part affected by the aura. Hence we may be instructed to look for the exciting cause of the disease, in cases which are characterised by the Aura epileptica, rather in the general state of the constitution and principal functions, than in any local irritation in the part whence the aura arises."

We shall only remark, that, at this period of life, in the human and many other subjects, epilepsy is not uncommon, and frequently ceases spontaneously as the new condition of the animal is completed. It is true, this is not always the case, and there is every reason to believe that Dr. Prichard's treatment was serviceable to his young patient.

The next case shews that the disease, occurring at this time of life, does not always cease spontaneously, as the patient was troubled with fits from fourteen to twenty-two years of age. The same plan was adopted, with some additional remedies, which last the writer conceives to have been of very little service. The patient had continued well from the end of November, 1814, to the 10th of January, 1815, and, as the author has good reason to believe, till the July following. There is, however, a little ambiguity in the dates, which, in a paper of this kind, should have been cautiously avoided.

The 3d case commenced about sixteen, and had continued eleven years, when Dr. Prichard undertook the cure, which in this instance also proved successful.

 The

The last is less decisive, because the patient, a female, had, on a former occasion, enjoyed an intermission of paroxysms for three years out of eight.

The paper concludes with some very just remarks on a subject we have often brought before our readers, and on which we gladly avail ourselves of the concurrence of so ingenious a writer.

"Before dismissing the subject of epilepsy, I wish to mention a suspicion which I have long entertained, that the accurate discrimination of convulsive diseases, as well as of many others, has been much impeded by the great desire which has prevailed of late years to simplify the nomenclature, and establish systematic notions concerning the nature and causes of distempers. I am persuaded that we commonly include under the name of epilepsy, diseases which differ in important circumstances. I am not, however, prepared at present to propose an arrangement of them which may be satisfactory to the public or to myself, but have only to offer a few cursory remarks. I would propose to limit the appellation of epilepsy to those cases which are accompanied with signs of determination of blood to the head, and to distinguish those convulsive affections attended with loss of consciousness, which arise from indigestion, worms, and other irritations not immediately affecting the head, by Sauvages' old name of Eclampsia."

It is not improbable, as the author's information becomes the more extensive, we may find his difficulties increase. However this may be, he has said enough to shew the danger of that alternate confidence and indecision with which nosology must, at different times, inspire or perplex a young practitioner. After this remark, however, we think it right to add, that epilepsy is, in our opinion, a disease sufficiently marked to admit a precise definition, and consequently a precise name.

Art. XIII.—*Case of painful Subcutaneous Tubercle.* By MARSHALL HALL, M.D. &c.

This is a very useful little case, and, with several others which have occurred, should teach every practitioner to be very minute in the examination of the part in which a patient complains, and also very attentive to the patient's history of his sufferings. By the removal of a very small tumour near the surface, an inconvenience was removed, which had for a long period interrupted the tranquillity and ease of the patient.

Art. XIV.—*Case of Disease in the Brain, with Pathological Observations.* By THOMAS SALTER, Member of the Royal College of Surgeons in London.

This paper is valuable, as adding to the number of facts which which may hereafter enable us to ascertain something concerning the nosology of the brain. The patient, without any cause which could be traced, but inflammation arising from exposure to wet and cold, after various symptoms about the eyes and other parts of the head, with proofs of cerebral oppression, at length died, worn out with disease. On examining the brain, besides effusion and other marks of inflammation, the pons varolii "had undergone great change in structure. A considerable portion of it was converted into a substance of a fleshy appearance, forming on its substance two triangles, with their apices nearly united; the base of one extending some way into the right crus cerebri; that of the other not only as far as the commencement of the medulla oblongata, but a little way upon it. The diseased part was not elevated above the general surface of the tuber annulare, but penetrated its substance to a considerable depth. It was very firm, and somewhat fibrous, though it cut much harder than muscular fibre. When detached from the other parts, which were soft and flabby, and easily squeezed from the diseased portion with the fingers, it was found to weigh two drachms. To preserve it, I put it into muriatic acid, at the same time I doubted the propriety of the use of this fluid, considering how widely different the parts were, not only from healthy brain, but also from that organ under any disease with which I had been before acquainted. However, I must acknowledge myself to have been surprised on finding, in a few hours, a great deal of it dissolved, and that, by the next day, the whole had undergone complete solution, the acid remaining clear and transparent as before, except being a little heightened in colour."

Some pathological remarks follow, with hints which, for the reason given by the author, we think it right to transcribe.

"I must regret, (says he,) that the ignorant prejudices of the patient's friends would not allow the abdomen to be examined, as I thought it a case likely to throw some light upon the subject of that reciprocal morbid sympathy which some distinguished physicians have supposed to exist between the brain and liver, and which I believe to be an opinion founded both on just principles and accurate observation. At the same time, I think, that, while the attention of the profession has of late been so much directed to the latter organ, the effects of diseases in the other viscera upon the brain, have not received that investigation which their importance would seem to merit. I was led to this conclusion, from the consideration of the origin and distribution of the abdominal nerves, and from having lately had an opportunity of examining, in my own practice, and in that of others, four patients who died of hydrocephalus hydrocephalus internus, in all of whom the liver was natural. In the two first there was no apparent disease in the abdomen. In the third, the spleen was greatly inflamed, and full of hard white tubercles, about half the size of peas. It adhered so firmly to the diaphragm, that its substance was torn in detaching it. The state of the spleen, in the fourth case, differed in no respect from the preceding, but the lungs were, I think, still more loaded with tubercles than the spleen, though many of them were smaller, resembling millet seeds. These were decided cases of hydrocephalus internus, such as no one, at all accustomed to see the disease, could mistake.

"I also lately dissected a case of apoplexia, where the liver and all the other abdominal viscera were found in a healthy state, though the patient had, for a long time previous to his death, been subject to a disordered state of the digestive organs. This, and the two first cases of hydrocephalus before-mentioned, would seem to warrant an opinion, that it is not at all times necessary to give rise to sympathetic disorganization in the brain, that actual diseased structure should exist in the abdominal viscera, and that sometimes a deranged action of the organs alone will be sufficient to produce that effect. A case but a short time ago occurred to me, which I think demonstrates the fact; and, as it appears to me an important one, I may, perhaps, whilst on the subject, be excused the additional length it will give this article, by only briefly relating it.

"Mrs. ———, a lady 49 years of age, rather below the middle stature, fair skin, dark hair, had been troubled for many years with dyspeptic and nervous complaints. The integuments had that peculiar sallow hue which usually attends cachectic diseases. Her indisposition had created a particular dislike to animal food, and, for many months previous to her death, she refused it altogether, eating only, in a very sparing manner, of solid aliment of any kind, and subsisting almost entirely upon milk and water. The bowels were in a very irregular state; sometimes confined, at others greatly relaxed; stools seldom healthy, often dark and extremely fetid, though mostly green and slimy, resembling the alvine evacuations of children suffering under constitutional irritation. The urine was secreted in proper quantity, but uniformly pale, and without sediment, not unlike the urine of the hysteric paroxysm. There was often pain in the back, between the scapulæ, and tenderness, with a degree of pain in the epigastrium; there was also a fullness in the right hypochondrium, though the liver could not be distinctly traced below the margin of the ribs; pulse quick and feeble, rarely below 100; tongue mostly clean, but chapped. This may be considered as a correct account of the patient's state, (the last fortnight excepted) for at least ten months before her death. She took the blue pill, and a variety of vegetable bitters, combined with the fixed and volatile alkalies, which, as long as they were continued, never failed to correct, in a considerable degree, the morbid condition of the abdominal viscera, and to alleviate all her symptoms.

symptoms. About a fortnight before her death, another and a more alarming train of symptoms made their appearance. The sickness, which was before only occasional, became now almost continued, though nothing but a little frothy mucus was brought up. She complained of indistinct vision, and was very lethargic; respiration became slow, not averaging more than twelve in the minute. The pulse also fell to eighty. She manifested the greatest disinclination to take any kind of sustenance, much persuasion on the part of her friends being often necessary to induce her to make the attempt to swallow. Scarce any urine was now secreted, but its character remained unchanged. There were tremulous motions of the voluntary muscles, especially when an attempt was made to exert them. Her skin was also at this time universally affected with prurigo. These symptoms continued with but little variation, until a few hours previous to the closing scene, when, as is usual in diseases of the brain, the pulse again rose, the artery beating an hundred in the minute. In about twenty-four hours after death, the body was examined. There was much fluid between the convolutions of the brain, under the tunica arachnoides. The veins of the pia mater were turgid, and numerous bloody points dotted the cut surface of the cerebrum. Two ounces of fluid were found in the lateral ventricles. The plexus choroides was pale, and the substance of the brain was very soft. On opening the abdomen, with an expectation to find disease in that cavity, the liver first struck me as being enlarged; but, as no diseased appearance was to be seen on any part of its surface, and as sections of its substance displayed no morbid state of the internal structure, I entirely gave up the notion, and consider that viscus to have been in the most natural state. The gall-bladder was two-thirds full of healthy bile. In the villous coat of the stomach were a few of those vascular spots described by Dr. Yellowly in the fourth volume of the Medico-Chirurgical Transactions, and which that writer does not consider as a mark of disease. I had often observed this appearance in the inner membrane of the stomach in persons who died of different diseases; and once in the greatest degree in a woman who was burnt to death, and who had no previous complaint, which I was unable to account for, until I had read that learned gentleman's ingenious paper upon the subject. All the other viscera were natural."

---

### MEDICAL AND PHILOSOPHICAL INTELLIGENCE.

*To the Editors of the London Medical and Physical Journal.*

